# Lamotrigine Reduces Stress Symptoms of Chronic Anxiety in the Times of the Covid-19 Natural Catastrophe-A Case Report

**DOI:** 10.3389/fpsyt.2021.655079

**Published:** 2021-06-21

**Authors:** Thuylinh L. Pham, George P. Chrousos, Andreas Merkenschlager, Katja Petrowski, Enrico Ullmann

**Affiliations:** ^1^Department of Child and Adolescent Psychiatry, Psychotherapy, and Psychosomatics, University of Leipzig, Leipzig, Germany; ^2^University Research Institute of Maternal and Child Health and Precision Medicine, National and Kapodistrian University of Athens, Athens, Greece; ^3^School of Medical Biology, South Ural State University, Chelyabinsk, Russia; ^4^Department of Neuropediatrics, University of Leipzig, Leipzig, Germany; ^5^Department of Medical Psychology and Medical Sociology, Clinic and Policlinic for Psychosomatic Medicine and Psychotherapy, University Medicine Mainz, Mainz, Germany; ^6^Department of Internal Medicine, Technical University of Dresden, Dresden, Germany

**Keywords:** Post-traumatic stress disorder, lamotrigine, inflammation, self-harm behavior, obesity

## Abstract

The SARS-CoV-2 pandemic has been a worldwide chronic, stress-inducing natural catastrophe associated with increased emotional challenging. Patients with Post-traumatic stress disorder (PTSD), self-injury behavior, and obesity are predisposed to aggravation of their symptoms at this time, requiring new therapeutic approaches to balance their disrupted neuro-hormonal stress axis. Here we present our observations of an off-label treatment with lamotrigine in an adolescent girl with PTSD, self-injury behavior, and obesity. Lamotrigine was an efficacious pharmaceutical intervention that helped the patient deal with chronic stress and associated anxiety. The results are discussed based on our previous basic research outcomes in animals and humans that focused on the glutamate-cortisol circuits within the limbic brain.

## Introduction

During the current war between humanity and SARS-CoV-2, new treatment options are needed to confront its adverse psychological consequences in patients with chronic stress-related disorders. Especially Post-traumatic stress disorder (PTSD) seems to be serious in the Covid-19 natural catastrophe (1). “Stress” is a state of disturbed homeostasis or “dyshomeostasis,” when stressors are poorly confronted by our adaptation mechanisms. Here we refer to the concepts of “cacostasis,” when disturbed homeostasis allows survival at the expense of good health, and “hyperstasis” (2), when homeostasis is associated with increased psychosomatic resilience to stressors. Generally, cacostasis is associated with a hyperactivation of the Limbic-Hypothalamic-Pituitary-Adrenal (LHPA) axis and un-needed and damaging systemic inflammation (“para-inflammation”), while hyperstasis is linked to decreased set-points of reactivity to stressors, and restrained activation of the LHPA axis and the inflammatory reaction (3).

We have shown that obese humans, labeled as low cortisol reactors, consume less food, and that more active and “offensive” rats have low levels of anxiety and increased glutamate+glutamine levels in the striatum and inversely related glutamate levels in the amygdala (3–5). These pathophysiological limbic circuit changes are present not only in patients with obesity and/or less physical activity, but also in patients with PTSD (6). The novel diagnosis of “complex PTSD” (CPTSD) was introduced in the new WHO diagnostic nomenclature (ICD-11), referring to a distinct subgroup of PTSD patients, who have experienced multiple and sustained traumas and have greater functional impairment than those with classic PTSD, mainly manifesting chronic anxiety (7). CPTSD includes disruptions in eating as well as non-suicidal self-injury behavior (8).

First-line interventions focusing specific symptoms included emotion regulation strategies, narration of trauma memory, cognitive restructuring, anxiety and stress management, and interpersonal skills. Meditation and mindfulness interventions were shown as an effective second-line approach for emotional, attentional, and behavioral (e.g., aggression) disturbances (9). To date no pharmaceutical interventions are available to treat PTSD adequately. Glutamate seems to be an important moderator in PTSD, while lamotrigine (LTG), a glutamate release-inhibitor, has been proposed as a new treatment option in PTSD (10). LTG is a member of the class of 1,2,4-triazines, in which the triazene skeleton has substitutions by amino groups at positions 3 and 5, and by a 2,3-dichlorophenyl group at position 6. LTG use is allowed in children and youths (>12 years of age) only in epilepsy therapy, and in adults in epilepsy-, myoclonus-, bipolar disorder-, and recurrent depression therapies. In adults, PTSD symptom reduction of around 50% occurred with LTG treatment compared to 25% reduction in the placebo group, while a significant weight reduction took place in obese patients taking LTG (11, 12). Based on our basic research results, we hypothesized that adolescents with PTSD and/or obesity, LTG will decrease CPTSD symptomatology and circulating inflammation markers, and will reduce self-injury behavior, as well as their Body Mass Index (BMI).

## Case Description

Our patient was a 17-year-old girl, living in a children's home far from her parents. She initially presented in Child and Adolescent Psychiatry in 2015, with self-injury behavior and suicidal thoughts. She reported that she was bullied at school, had anxiety, and had to change school. She was started on psychotherapy, which was continued for 2 years. Since 2018, shortly after she moved out from her mother's home to a children's facility, the patient received in- and out- patient care at the Departments of Child and Adolescent Psychiatry and Psychotherapy at Merseburg and the University of Leipzig (Germany, Table 1). After several crisis interventions in both departments and a suicide attempt by paracetamol uptake on the 18th of January 2020, we intensified the clinical diagnostics during her in-patient care from 22nd to the 26th of January 2020 and diagnosed complex PTSD presenting with self-injury behavior, suicidal ideation, recurrent depressive episodes, and overweight/obesity (BMI: 29.7 kg/m^2^). Furthermore, we found increased inflammation markers (circulating C-reactive protein and tumor necrosis factor-alpha, without an increase of interleukin 6, Table 2), which we interpreted as a result of chronic stress (13).

**Table 1:** Timeline of in- and out-patient care with the related diagnoses.

**Time range of in-patient stay**	**Clinic**	**Diagnoses**
May–August 2018	Clinic Merseburg	F33.2 Major depressive disorder, recurrent severe without psychotic features F44.9 Dissociative and conversion disorder, unspecified
December 2018	University Clinic Leipzig	F32.1 Major depressive disorder, single episode, moderate X84 Intentional self-harm by unspecified means
November 2019	University Clinic Leipzig	F33.2 Major depressive disorder, recurrent severe without psychotic features X84 Intentional self-harm by unspecified means
January 2020	Clinic St. Georg Leipzig	F39.1 Unspecified mood [affective] disorder F21 Schizotypal disorder
January–February 2020	University Clinic Leipzig	F43.1 Post-traumatic stress disorder

**Table 2:** Chronic stress measurement via psychometric and biological markers.

		**Date of chronic stress measurement**				
		**28th January 2020**	**26th February 2020**	**27th April 2020**	**6th October 2020**	**2nd December 2020**
Method of chronic stress measurement	SDQ	20	16	8	8	15
	SSS-8	13	10	3	3	12
	PHQ-9	21	8	6	4	21
	GAD-7	15	4	4	5	14
	PCL-5	42	22	14	11	41
	B-criteria for PTSD	11	8	4	4	6
	C-criteria for PTSD	4	3	1	1	5
	D-criteria for PTSD	17	4	3	2	17
	E-criteria for PTSD	10	7	6	4	13
	Body mass index (BMI)	29.7	29.4	29.1	27.22	26.5
	Weight in kg	68.2	72.2	68.8	65.4	63.7
	Size in cm	153	153.8	154	155	155
	Systolic arterial pressure in mmHg	90	128	137	118	125
	Diastolic arterial pressure in mmHg	65	83	91	70	85
	Mean arterial pressure (MAP)	73.3	98	106	86	98
	Heart rate in beat/minute (BPM)	127	86	120	80	85
	Tumor Necrosis Factor Alpha		9.0 pg/ml	<8.1 pg/ml		
	c-Reactive Protein (crp)	11.47 mg/l	19.6 mg/l	13.76 mg/l	11.79 mg/l	8.11 mg/l
	Interleukin-6		<1.5 pg/ml	<1.5 pg/ml		

*SDQ, Strengths and Difficulties Questionnaire; SSS-8, Somatic symptom scale-8 items; PHQ-9, Patient Health Questionnaire- 9 items; GAD-7, Generalized Anxiety Disorder Scale- 7 items; PCL-5, PTSD-Checklist for DSM 5; Lamotrigine treatment was started on 19th February 2020 with 25 mg/day and reaching the final dose (200 mg/day) at 20th April 2020. Recurrent self-injury behavior until August 2020. No self-injury behavior in September, October, and November 2020. Negative Sars-CoV-2 PCR (E-Gen) pharyngeal on 13th April, 13th May, and 28th July 2020*.

Due to recurrent psychiatric crises leading to longer episodes of in-patient care within our department of Child and Adolescent psychiatry, we used several treatment options (emotion regulation strategies, narration of trauma memory, cognitive restructuring, anxiety and stress management, and interpersonal skills). These therapeutic approaches, based on the consensus strategy of the International Society for Traumatic Stress Studies Complex Trauma Task Force (ISTSS), were not sufficiently effective (9).

Thus, we decided to start a new off-label treatment option with lamotrigine (LTG) based on evidence in adult subjects. Using the glutamate-moderating effects of lamotrigine, we hypothesized a beneficial role in improving PTSD symptomatology, including self- aggressive (externalizing) and depressive (internalizing) behavior, and a potential reduction of the inflammation markers and BMI. After about 8 months of treatment with LTG, the patient herself decided to stop taking this medication, so we began tapering lamotrigine on the 14th October 2020.

The dosage schedule of lamotrigine was as follows starting at 19th February 2020:

Week 1, 2: 0-0-25 mg
Week 3, 4: 0-0-50 mg
Week 5, 6: 50mg-0-50 mg
Week 7, 8: 50mg-0-100 mg
Week 9, 10: 100mg-0-100 mg
(After week 34: 50-mg-0-50 mg)

### Diagnostic Assessment and Statistical Analysis

Besides a continuous evaluation by a consultant in child and adolescent psychiatry and psychotherapy, we used our psychometric diagnostic trauma inventory (Table 2) focusing on specific PTSD symptoms: Lifetime incidence of traumatic events was assessed using the LEC-5 (14), a 17-item self-report screening instrument recording traumatic events in accordance with the DSM-5 A criterion of PTSD. PTSD symptoms were measured using the 20-item Posttraumatic Stress Disorder Checklist for DSM-5 [PCL-5; (14)], which covers the four PTSD symptom clusters rated on a five-point scale. Depression symptoms were measured with the 9-item Patient Health Questionnaire (PHQ-9; four-point scale) (15), whereas symptoms of anxiety as well somatic symptoms were assessed using the 7-item Generalized Anxiety Disorder Scale (GAD-7; four-point scale) (16) and the 8-item Somatic Symptoms Scale (SSS-8; five-point scale) (17), respectively. Emotional and conduct problems in the last 6 months were assessed using the 25-item Strength and Difficulties Questionnaire (SDQ; three-point scale) (18). All scales provided sufficient to excellent internal consistencies (αs between 0.68 and 0.92).

We measured height and body weight, blood pressure, and heart rate, while we monitored the status of self-injury behavior by body checks. As there are known side effects from changes in the activity of the limbic-brain stress axis, we closely monitored inflammatory status, especially after initiation of LTG therapy on the 19th of February 2020. No other medications were taken by the patient. During treatment, we measured circulating C-reactive protein, interleukin-6 and tumor necrosis factor-α. All chronic stress parameters were progressively reduced during the LTG treatment, including body weight and inflammation markers (Table 2). All measurements were used 4 weeks before, in the course of and after the LTG treatment (Table 2).

For statistical analysis, the mean square were generated by using the data of 4 measurements (before and after beginning the treatment with lamotrigine) of the SSS-8, PHQ-9, GAD-7, PCL-5, body mass index (BMI), mean arterial pressure (MAP), heart rate in beat/minute (BPM), c-reactive protein (crp); a = computed using alpha = 05; *F*_(3;39)_ = 3.73, *p* = 0.2, power = 77%; respecting a possible injury of the sphericity (df-correction via Greenhouse-Geisser) the power is 61% (*p* = 0.04).

### Ethics Statement

The patient was 17 years of age at the beginning of the study and gave a signed informed consent together with the legal guardian according to the description of the off-label used study with LTG. Moreover, on 25 January 2021, when the patient was already 18 years of age, she gave once again a signed informed consent for the publication of any potentially identifiable images or data included in this article.

## Discussion

Using the glutamate modulator LTG, we showed a reduction of stress-related symptoms, including self-injury behavior, as well a body weight reduction, effects expected and substantiated by our previous basic research results in humans and animals. Moreover, after reducing the treatment dose of LTG, a recurrence of the focal symptoms took place, which is in line with previous findings (10–12).

The mechanism of the moderating role of glutamate within the limbic stress system circuits is largely unknown, however, lower glutamate levels in the amygdala of more active subjects seems to be associated with less anxiety and decreased plasma cortisol (CORT) levels (3). On the other hand, lower glutamate levels in the striatum were associated with lower adrenal 11-dehydrocorticosterone and higher plasma CORT (5). These inverse reaction patterns of the limbic Glu-Cort circuits could be explained by the paradigms of caco- and hyper-stasis (2). We assume that cacostasis here is associated with chronic activation of regulatory systems away from their normal state of operation, to finally establish a new “lower” set point of responsiveness to stressors, while the inverse takes place in hyperstasis. Thus, we hypothesize that more active subjects reach faster higher hyperstatic set points, that the inactive ones, via chronic physical activation.

The behavioral outcomes of the above inverse LHPA chronic stress reactions are unknown. Regarding the eating behavior of chronically stressed obese subjects indicates that hyperstatic scenarios lead to a less food intake (4). This could be similar to behavioral changes related to PTSD that should be examined in future studies.

The strengths of our approach in our adolescent patient with PTSD are the continuous evaluation and monitoring of the case by a consultant in child and adolescent psychiatry and psychotherapy, underpinned, in parallel, by our standardized psychometric inventory. Moreover, we focused not only on psychometric data, but also on anthropometric data, inflammation markers, and self-injury behavior, as indicators of CPTSD, while we minimized side effects from a potential SARS-CoV-2 infection. Our sample of one precludes any measure of a useful effect size and does not allow any general conclusions, however, earlier data indicated similar results in adults. Thus, LTG-treated patients showed improvement in PTSD symptoms compared to placebo, as well a significant body weight reduction (10–12). For patients with non-suicidal self-injury behavior diagnosed, for instance, with a Borderline Personality Disorder (BPD), limited data are available, indicating no clinical effectiveness of LTG in this condition (19). We interpreted these differences of treatment benefits between PTSD and BPD as an apparent marker for the clinical distinction of these two disorders, which should be considered, especially as the new diagnosis of “CPTSD” has been introduced within the new ICD-11 nomenclature (7, 8).

From this case we can conclude that LTG is a potentially efficacious therapeutic approach in patients with PTSD with/without self-injury and disruptions in eating behavior leading to overweight/obesity.

### Patient Perspective

Chronic stress scenarios with phenotypic psychological and emotional challenges are to be expected in the course of the current COVID-19 pandemic. PTSD/ CPTSD, as well as self-harm and disruptions in eating behavior, are just a few diagnostic examples. Mid- and long-term psychotherapeutic approaches can be supported by a temporary LTG treatment leading to a damping and/or balancing of the stress system dyshomeostatic set-points spanning the range from caco- to hyper-stasis. More evidence is needed to translate our basic research results to humans, and large cohorts of patients should be studied in placebo-controlled pharmaceutical trials.

## Data Availability Statement

The raw data supporting the conclusions of this article will be made available by the authors, without undue reservation.

## Ethics Statement

Ethical review and approval was not required for the study on human participants in accordance with the local legislation and institutional requirements. Written informed consent to participate in this study was provided by the participants' legal guardian/next of kin. The patient was 17 years of age at the beginning of the study and gave a signed informed consent together with the legal guardian according to the description of the off-label used study with LTG. Moreover, at 25 January 2021, when the patient was already 18 years of age, she gave once again a signed informed consent for the publication of any potentially identifiable images or data included in this article.

## Author Contributions

EU and AM organized the study, analyzed the data, drafted the manuscript, and prepared the figures and tables. TP and EU collected the samples and analyzed the data. KP and GC provided intellectual and scientific input and data analyzes and interpretation. All authors reviewed the manuscript.

## Conflict of Interest

The authors declare that the research was conducted in the absence of any commercial or financial relationships that could be construed as a potential conflict of interest.
